# Chemical Composition, Antioxidant Activity and Cytocompatibility of Polyphenolic Compounds Extracted from Food Industry Apple Waste: Potential in Biomedical Application

**DOI:** 10.3390/molecules28020675

**Published:** 2023-01-09

**Authors:** Parinaz Hobbi, Oseweuba Valentine Okoro, Maryam Hajiabbas, Masoud Hamidi, Lei Nie, Véronique Megalizzi, Paul Musonge, Gianina Dodi, Amin Shavandi

**Affiliations:** 1 École Polytechnique de Bruxelles, Université Libre de Bruxelles (ULB), 3BIO-BioMatter, Avenue F.D. Roosevelt, 50-CP 165/61, B-1050 Brussels, Belgium; 2 Laboratory of Pathophysiological and Nutritional Biochemistry, Faculty of Medicine, Université Libre de Bruxelles, 808 Route de Lennik, Blg G/E CP 611, B-1070 Brussels, Belgium; 3 Department of Medical Biotechnology, Faculty of Paramedicine, Guilan University of Medical Sciences, Rasht 41887-94755, Iran; 4 College of Life Sciences, Xinyang Normal University (XYNU), Xinyang 464000, China; 5 Pharmacognosy, Bioanalysis & Drug Discovery Unit, Faculty of Pharmacy, Université Libre de Bruxelles, B-1070 Brussels, Belgium; 6 Institute of Systems Science, Durban University of Technology, Durban 4000, South Africa; 7 Faculty of Engineering, Mangosuthu University of Technology, Durban 4000, South Africa; 8 Advanced Centre for Research-Development in Experimental Medicine, Grigore T. Popa University of Medicine and Pharmacy of Iasi, 700115 Iasi, Romania

**Keywords:** apple pomace, polyphenolic compounds, waste valorization, antioxidant activity, biological properties, cytocompatibility

## Abstract

Apple pomace (AP) from the food industry is a mixture of different fractions containing bioactive polyphenolic compounds. This study provides a systematic approach toward the recovery and evaluation of the physiochemical and biological properties of polyphenolic compounds from AP. We studied subcritical water extraction (SCW) and solvent extraction with ethanol from four different AP fractions of pulp, peel, seed, core, and stem (A), peel (B), seed and core (C), and pulp and peel (D). The subcritical water method at the optimum condition resulted in total polyphenolic compounds (TPC) of 39.08 ± 1.10 mg GAE per g of AP on a dry basis compared to the ethanol extraction with TPC content of 10.78 ± 0.94 mg GAE/g db. Phloridzin, chlorogenic acid, and quercetin were the main identified polyphenolics in the AP fractions using HPLC. DPPH radical scavenging activity of fraction B and subcritical water (SW) extracts showed comparable activity to ascorbic acid while all ethanolic extracts were cytocompatible toward human fibroblast (3T3-L1) and salivary gland acinar cells (NS-SV-AC). Our results indicated that AP is a rich source of polyphenolics with the potential for biomedical applications.

## 1. Introduction

Food industry waste is mainly generated by fruit processing units in the form of pomace, which is recognized by its high acidity, water content, and fast spoilage rate [1]. Notably, AP is a major source of fruit waste with up to 12 million tons per year globally, which is comprised of apple peel, pulp, seeds, cores, stems, and their mixture [2] produced mainly after the juicing process via a series of steps such as milling, liquefaction, and juice extraction [3].

Due to poor nutritional value, AP waste has not been recognized as a suitable material for animal feedstock or land fertilization and is usually released into the environment, leading to pollution issues [4]. Therefore it is crucial to explore strategies for value recovery, following the circular economy paradigm [5]. AP contains polyphenolic compounds that may function as natural antioxidants and antimicrobial, anti-cancer, and anti-diabetic agents and thus present considerable potential for pharmaceutical, cosmetic, and food applications [6,7]. Polyphenolic compounds from AP extracted via organic solvents including methanol, ethanol, and acetone have demonstrated various desirable biological properties [8]. For instance, Bai et al. showed a radical scavenging rate of 90.96 ± 10.23%, ABTS radical scavenging of 89.78 ± 6.54%, and a strong ferric reducing power of 8.30 ± 0.71 µmol Trolox equivalents kg^−1^ dry AP, for the tested AP [9]. The antibacterial activity of AP extracts against various species of *Escherichia coli*, *Staphylococcus aureus*, and *Salmonella enterica* was also reported [10]. The feasibility of AP polyphenolic compounds for utilization in topical formulations was highlighted by Suárez et al., who reported more than 50% antiviral activity for the AP polyphenolic extract against replication of HSV-2 and HSV-1 in the tested Vero cells [11]. The potential application of polyphenolic compounds extracted from various fruit wastes as well-known bioactive compounds has been well recognized for the fabrication of polyphenolic-based biomaterial for different biomedical applications. For example, a porous composite scaffold fabricated by combining mangosteen (*Garcinia mangostana*) peel polyphenolic extract and collagen showed its potential use in bone tissue regeneration because of the polyphenol’s effect on collagen stabilization, mineral (hydroxyapatite) deposition, and the prevention of free radical oxidation [12]. Another study demonstrated that iron oxide nanoparticles prepared and stabilized by green tea leaf polyphenolic extract could be a potential biomaterial for cancer drug carriers [13]. Polyphenolic extract from pomegranate peel displayed its potential in wound healing through the enhanced proliferation and migration of human dermal fibroblast (HDF) cells and its positive influence on cell mechanisms necessary for the wound healing process [14].

Organic solvents such as ethanol, acetone, and methanol, and SCW technology as a green technique have been used for the extraction of polyphenolics from different food wastes [15,16,17]. However, the efficiency of these methods on a single industrial food waste has not been broadly evaluated and the importance of waste fractions, e.g., seeds and the core of the pulp, have not been investigated. Therefore, the current research seeks to employ two extraction approaches for polyphenol compound recovery from different fractions of AP. In this study, the response surface method (RSM) was applied to explore the effect of parameters such as time, temperature, solid-to-solvent ratio, and particle size on the efficiency of SCW for the extraction of polyphenolics. We also for the first time explored four different fractions of AP for polyphenolic extraction and unlike in existing studies, a comprehensive investigation of the biological properties of different polyphenolic extracts was undertaken. We used HPLC analysis to quantify and identify the extracted polyphenolics and measured the antioxidant activity of the extracted compounds based on DPPH radical scavenging activity (% *D_IA_*). Furthermore, the cell biocompatibility of the ethanolic extracts from AP fractions was evaluated.

## 2. Results and Discussion

### 2.1. Physicochemical Determination of the Apple Pomace Fractions

The physicochemical characterization of different fractions of AP is depicted in Table 1. Fraction B and fraction D contained the highest carbon and oxygen contents, respectively. Sulfur constituted the lowest element in all the fractions. The elemental analysis results were found to be comparable to those reported in previous studies with some differences [18]. For example, fraction C had a higher nitrogen value compared to the value ranges of 0.42–1.70% *w/w* achieved in studies by Gowman et al. and Verma et al. [19,20], which can be related to the high protein content of apple seed as found by Vidović et al. [21].

The observed differences can be correlated to different apple varieties (cultivars), their origin, and the effect of processing techniques [8,22]. For instance, the AP used by Gowman et al. was from an apple juice processing company that processed a mixture of different varieties of apples (Northern Spy, MacIntosh, Empire, Gala, Ambrosia) with several steps of air drying [19], and in a study by Verma et al., the AP was the waste of apple juice from the Malus Domestica variety [20]. The APs used in the present study were obtained from three varieties of Belgian apples including Reinette Hernaut, Cox, and Topaz, which were utilized for the production of apple puree and apple chunks. Fraction B contained a peel of AP derived from the production line of apple chunks subjected to steam injection (130 °C) for a few seconds. Fraction C was also the waste of the production line of apple chunks but comprised seed and core wastes.

Fraction D and fraction C contained the highest and lowest moisture, respectively (Table 1). The difference in moisture content of the fractions is attributed to the different processes applied in the production lines for apple puree and apple chunks. Fraction A was from the production line of apple puree, and other fractions were obtained from apple chunk production. The high moisture content of fractions D and B can be due to the washing step after peeling and injection of steam.

Carbohydrates were the most abundant chemical compound in all fractions ranging from 57.54 ± 5.15% *w/w* to 71.94 ± 1.30% *w/w*. The highest carbohydrate content was obtained in fraction A and fraction D, which could be due to the presence of pulp consisting of a mixture of simple sugars such as glucose, fructose, and sucrose as well as organic acids in these fractions [23].

The lignin content was within the range previously reported in different AP samples (8.87–29.16% *w/w*) [19]. Fraction C contained a higher protein content compared to other fractions, which was not within the range (1.2–6.91% *w/w*) achieved for the protein content of different AP samples in previous studies [24]. This observation can be correlated to the high protein content in apple seeds [21]. The high lipid content in fraction B was not within the range (0.26–8.49% *w/w*) found in the previous research for AP [24] and the value of 9.96 ± 1.52% *w/w* obtained from apple peel [25]. Ash, which represents the content of inorganic compounds and main minerals [19], has been reported within the range value of 0.5–6.10% *w/w* in the literature [26]. Fraction C contained the highest ash content, which can be correlated to the high content of minerals in the apple seed and core. The studied fractions of AP can be a potential source to produce different valuable materials such as biochemicals, biofuels, biopolymers, and bioactive and functional compounds.

### 2.2. Total Polyphenolic Compounds

#### 2.2.1. Ethanol Extraction

The TPC of the ethanolic extracts of AP fractions is shown in Table 2. As the data show, the mixture of pulp and peel (Fraction D) contained higher TPC content than other AP fractions. Several studies, however, reported higher TPC of peel compared to other fractions of AP [27,28]. For example, the peel of different varieties of apple including Golden Delicious, Idared, Rome Beauty, and Cortland contained the highest TPC compared to other fractions of a mixture of peel and pulp and pulp alone [28]. Similarly, the peel extract of an Italian apple (Pelingo) showed higher TPC content than pulp [27]. The observed differences in the TPC content may be due to the cultivation environment, cultivar, used apple fractions (e.g., pulp), the apple processing technology, and the method of polyphenol extraction [29]. For instance, the ultrasound-assisted extraction resulted in higher TPC contents of 3.68 ± 0.20 mg/g db and 1.46 ± 0.19 mg/g db of Papierowka and Gold Milenium extracts, respectively, compared to those obtained with conventional extraction (80% ethanol), having TPCs of 2.02 ± 0.13 mg/g db and 1.15 ± 0.04 mg/g db [30]. A summary report of differences in TPC results of several studies in comparison with our results is provided in Table 2.

The result of the current work also exhibited the presence of high polyphenols in the mixed seed and core fraction. The seed, which constitutes 2–3% of the total mass of AP, has been shown to contain various polyphenolic compounds. Gunes et al. showed that the TPC content was different among apple cultivars, and the extract of defatted seed flour of the Super Chief cultivar contained the highest content of TPC (5.14 mg GAE/g db) [31]. Considering the highest TPC content of fraction D using ethanol extraction in the present study, this fraction was selected for the SCW optimization study (Section 2.2.2).

**Table 2:** A summary of the previous reports on the total polyphenolic compounds (TPC) content that was obtained from different types of apple pomace (AP).

Type of AP	Cultivar	Extraction Method	TPC (mg GAE/g db)	Ref
Peel, pulp, core	Gold Milenium, Papierowka	Ethanol:water (80:100, *v/v*), sample/solvent (1:25 *w/v*), three steps extraction with solvent, 60 °C, 1.5 h	Gold Milenium: Peel = 0.17 Pulp = 0.04 Core = 0.09 Papierowka: Peel = 0.43 Pulp = 0.07 Core = 0.19	[30]
Peel, pulp, core	Guoguang, Fuji, Wanglin, Golden Delicious	Aceton:water (80:100, *v/v*), sample/solvent (1:4 *w/w*), 5 min, chill temperature	* Peel = 1.19 ± 0.02–1.7 ± 0.02 * Pulp = 0.65 ± 0.05–1.02 ± 0.07 * Core = 0.9 ± 0.03–1.36 ± 0.12	[32]
Seed	Golden Delicious, Red Delicious	Methanol:water (80:100, *v/v*), sample/solvent (1:10 *w/v*), room temperature, 30 min	Red delicious = 12.30 ± 0.96 Golden delicious = 7.17 ± 0.47	[33]
Seed	Fuji Zhen Aztec, Granny Smith, Pink Lady, Super Chief, Jeromine	Methanol:water (80:100, *v/v*), sample/solvent (1:10 *w/v*), room temperature, 30 min	Fuji Zhen Aztec = 2.86 ± 0.02 Granny Smith = 3.58 ± 0.05 Pink Lady = 4.1 ± 0.1 Super Chief = 3.62 ± 0.03 Jeromine = 5.14 ± 0.05	[31]
Whole fruit, peel	Red Delicious Starking, Golden Delicious, Granny Smith, Jona Gold, Royal Gala	Methanol: water (90:100, *v/v*), sample/solvent (2:1 *w/v*), chill temperature, 20 min, two steps extraction with solvent	* Peel = 1.56–4.00 * Whole fruit = 0.8–1.96	[34]
Peel	Rome Beauty	Aceton:water (80:100, *v/v*), sample/solvent (1:4 *w/w*), 5 min, chill temperature	* Fresh peel = 5.2 ± 0.14 * Air-dried blanched peel = 4.64 ± 0.27 * Freeze-dried blanched peel = 4.60 ± 0.42	[35]
Peel, pulp, pulp + peel	Cortland, Idared, Rome Beauty, Golden Delicious	Aceton: water (80:100, *v/v*), sample/solvent (1:4 *w/w*), 5 min, chill temperature, two steps extraction with solvent	Peel = 3.09 ± 0.32–5.89 ± 0.83 Pulp = 0.76 ± 0.04–1.03 ± 0.12 pulp + peel = 1.19 ± 0.15–1.59 ± 0.15	[28]
AP, industrial AP (Nectar)	Pinova, Reinders, Jonagold, Iduna, Braeburn	Methanol:water (80:100, *v/v*), sample/solvent (1:16 *w/v*) 150 min, room temperature	Pinova = 7.96 ± 0.37 Reinders = 8.67 ± 0.39 Jonagold = 8.53 ± 0.39 Iduna = 6.47 ± 0.31 Braeburn = 5.59 ± 0.25 Nectar = 4.22 ± 0.18	[36]
AP	Golden Delicious	Methanol, ethanol, acetone, ethyl acetate, chloroform sample/solvent (1:5 *w/v*), 37 °C, 40 min	Methanol = 3.05 ± 0.82 Ethanol = 2.87 ± 0.75 Acetone = 2.15 ± 0.35 Ethyl acetate = 2.51 ± 0.42 chloroform = 1.62 ± 0.23	[37]
Industrial AP Fraction A, Fraction B, Fraction C, Fraction D Industrial AP (Fraction D)	Reinette Hernaut, Cox, Topaz	Ethanol:water (50:50, *v/v*), sample/solvent (1:80 *w/v*), 20 min, 60 °C	Fraction A = 8.60 ± 0.26 Fraction B = 9.56 ± 0.22 Fraction C = 7.14 ± 0.29 Fraction D = 10.78 ± 0.94	Present study
		Subcritical water extraction, sample/solvent: (1:100 *w/v*), 75 min, 203.71 °C, mean sample particle size: 500 µm	Fraction D = 39.08 ± 1.10	Present study

* TPC = (mg GAE/g fresh weight basis). Fraction A (pulp, peel, seed, core, and stem), fraction B (peel), fraction C (seed and core), and fraction D (pulp and peel).

#### 2.2.2. Subcritical Water Extraction

The experimental results highlighting the TPC content of the SW extract of the AP fraction (fraction D) for different experimental conditions influenced by the four extraction factors of temperature, solid-to-solvent ratio, mean sample particle size, and time are presented in Table 3. Fraction D was utilized for SCW since it exhibited the highest TPC compared to other fractions with ethanol extraction (Section 2.2.1).

As the results exhibit (Table 3), the TPC content varied from 5.30 mg GAE/g db to 39.01 mg GAE/g db. The highest TPC was obtained at the conditions of extraction temperature, 220 °C; solid-to-solvent ratio, 1 g/100 mL; extraction time, 75 min; and mean sample particle size of 750 µm. The results revealed that the highest TPC content could be achieved at the highest temperature (220 °C) and the lowest solid-to-solvent ratio (1 g/100 mL), indicating that temperature and solid-to-solvent ratio are two crucial factors affecting the efficiency of SCW of TPC from AP. An empirical quadratic model was developed based on the experimental data (Table 3) that exhibit the dependence of the TPC content on the process parameters presented in (Equation (1));

(1) $$\begin{array}{c} Y=-76.7\;+\;0.986\;T\;+\;0.328\;t\;+\;0.0366\;S\;-\;2.84\;W\;-\;0.002243\;T^{2}\;-\;0.001415\;t^{2}\\ \;\;\;-\;0.000032\;S^{2}+0.1851\;W^{2}\;-\;0.000668\;T\times t\;-\;0.000050\;T\times S\;-\;0.01406\;T\times W\;\\ \;\;\;+0.000049\;t\times S\;-\;0.00695\;t\times W+0.00223\;S\times W \end{array}$$

The obtained R^2^ value (R^2^ = 0.9599) for the model indicated the sufficiency of the selected regression model for the description of the experimental results since the R^2^ value was greater than the value of 0.7, which is considered acceptable in scientific works [38]. The values of adjusted-R^2^ and predicted-R^2^ were achieved at 0.9132 and 0.7841, respectively, indicating that the model sufficiently describes the dependency of the response on process parameters and also provides reasonable predictions for new observations [39]. The Minitab software predicted the optimum TPC content to be 39.63 mg GAE/g db at temperature, time, mean sample particle size, and solid-to-solvent ratio values of 203.71 °C, 75 min, 500 µm, and 1 g/100 mL, respectively. The experimentally obtained TPC content at the optimum conditions was determined to be 39.08 ± 1.10 mg GAE/g db, showing comparable content to the predicted TPC content. The relative absolute deviation (RAD) was achieved at 1% less than 5%, which is the minimum acceptable RAD [38]. Therefore, the analysis of the experimental results demonstrated the validity of the experiment design employed in this study as well as the precision of the empirical model developed for SCW of TPC from AP. When the TPC content of AP extract using SCW technology is compared to that obtained with ethanol with values of 39.08 ± 1.10 mg GAE/g db and 10.78 ± 0.94 mg GAE/g db, respectively, we could conclude that SCW showed higher efficiency in the extraction of polyphenolic compounds from AP.

##### Independent Effects of Process Variables on TPC

**Figure 1:**
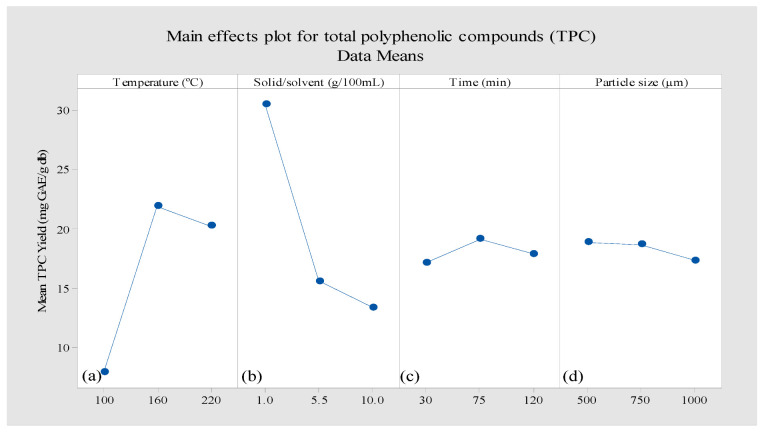
The independent effects of the process parameters of (**a**) temperature (°C), (**b**) solid-to-solvent ratio (g/100 mL), (**c**) time (min), and (**d**) mean sample particle size (µm) on TPC content of apple pomace extract using subcritical water extraction.

The independent effects of process parameters on TPC content were investigated using 2D plots (Figure 1). As the data indicate, the TPC content is highly influenced by the variation in two parameters, the solid-to-solvent ratio and the temperature, whose main effects are shown to be significant (*p* < 0.05). TPC increased from 7.91 to 21.93 mg GAE/g db by increasing temperature from 100 °C to 160 °C as shown in Figure 1a. This observation is expected since, in SCW processes, the temperature is considered the most crucial parameter due to its effect on the polar properties of water [40]. High diffusivity and low viscosity of subcritical water could also improve the solubility and mass transfer rate of polyphenols and their release from the solid matrix to the water [41]. Moreover, a high temperature (160 °C) could disrupt the interactions between polyphenolic compounds and polysaccharides or proteins, which could enhance the rate of diffusion and improve the rate of TPC extraction [15]. However further increments in the temperature from 160 °C to 220 °C led to a reduction of TPC content (20.25 mg GAE/g db), which can be correlated to the degradation of thermal-sensitive polyphenolic compounds exposed to high temperatures [42]. Furthermore, some polyphenolic compounds that are polymerized at high temperatures could reduce free polyphenols [43]. Studies associated with polyphenol extraction using SCW were typically performed at temperatures lower than 220 °C because of the probability of the degradation of polyphenolic compounds at temperatures higher than 200 °C [42,44].

Another parameter of the solid-to-solvent ratio exhibited a significant effect on TPC content. The highest TPC content of 30.51 mg GAE/g db was obtained at the lowest solid-to-solvent ratio of 1 g/100 mL (Figure 1b). The efficiency of the extraction in a static-mode SCW could be considerably affected by the solubility of polyphenolic compounds and the equilibrium concentration of the solid-to-solvent ratio [39]. In this study the right solid–solvent equilibrium concentration was provided at a solid-to-solvent ratio of 1 g/100 mL, resulting in enhanced solubility of polyphenolic compounds, which could positively affect the extraction rate and efficiency [45].

An increase in the ratio of solid-to-solvent (1 g/100 mL to 10 g/100 mL) caused a decrease in TPC content (from 30.51 mg GAE/g db to 13.41 mg GAE/g db), which could be due to the low solubilization of polyphenolic compounds as the concentration of AP sample in the extraction medium was increased (10 times) [39]. When the time was increased from 75 to 120 min (Figure 1c), TPC reduced from 19.15 mg GAE/g db to 17.9 mg GAE/g db, which may be due to the degradation of thermo-labile extracted polyphenolic compounds due to sustained heating [16].

Extraction of polyphenolic compounds can potentially reach higher efficiencies when smaller particle size is applied [16,17] as finer particle size with larger surface area reduces the diffusion distance between polyphenolic compounds and water, which resulted in enhancing diffusion and mass transfer of polyphenols [40]. The highest TPC content (18.90 mg GAE/g db) was achieved when the smallest mean sample particle size (500 µm) was employed (Figure 1d).

##### Interaction Effects of Variation in the Process Variables on TPC

**Figure 2:**
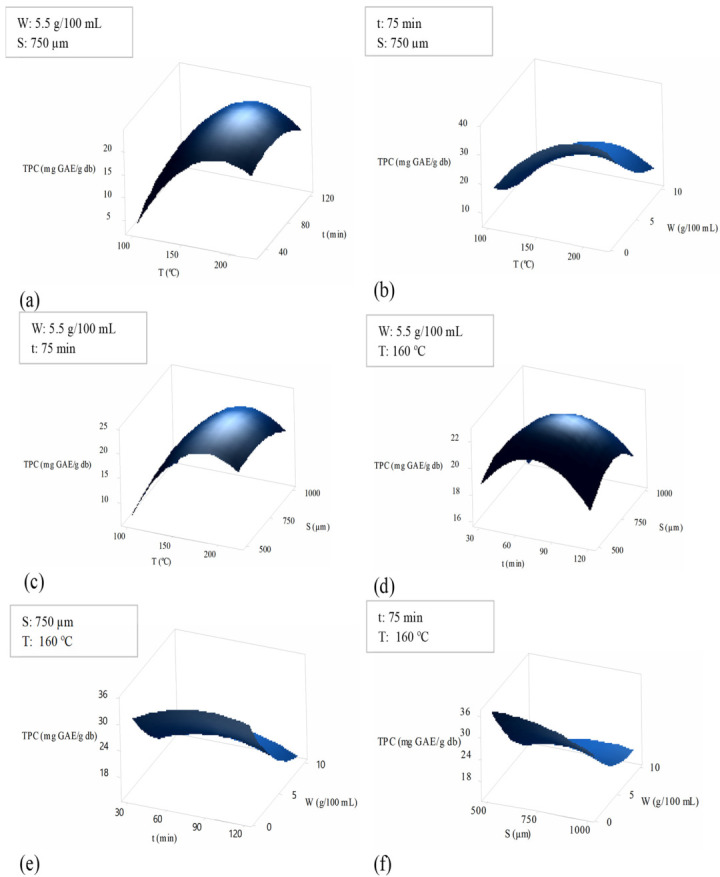
Interaction effects of the subcritical water extraction parameters on total polyphenolic compound (TPC) content represented by response surface plots. The values of the process parameters kept constant are highlighted in legends. T = temperature, t = time, W = solid-to-solvent ratio, S = mean sample particle size.

Three-dimensional (3D) plots were created to investigate the interaction effect of process variables on TPC content (Figure 2). The data were made by changing two factors within the experimental range while keeping two other variables at constant middle-level values.

The interaction effect of changes in temperature and time on TPC content exhibited that increasing temperature (from 100 °C to 200 °C) and time (from 30 min to 120 min) increased the TPC content (Figure 2a). In addition, at a temperature range from 160 °C to 220 °C, high TPC content (>15 mg GAE/g db) could be obtained, implying that extraction can be efficiently done in a shorter time by increasing temperature.

The interaction effect of changes in solid-to-solvent ratio and temperature on TPC content is depicted in Figure 2b, indicating an increase in TPC content with increasing temperature (from 100 °C to 220 °C) and decreasing solid-to-solvent ratio (from 10 g/100 mL to 1 g/100 mL). Notably, at a temperature range from 160 °C to 220 °C and a low solid-to-solvent ratio (1 g/100 mL), high TPC content (>35 mg GAE/g db) can be achieved. This observation emphasizes that the effect of decreasing the solid-to-solvent ratio and increasing temperature is favorable for TPC extraction efficiency.

The interaction effect of changes in temperature and mean sample particle size on TPC content is presented in Figure 2c. The data demonstrate that enhancing the temperature (from 100 °C to 220 °C) and mean sample particle size (from 500 µm to 1000 µm) led to an increase in TPC content such that the optimum TPC content (>21 mg GAE/g db) may be obtained at a temperature range of 160–210 °C and mean sample particle size range of 500–900 µm. The result depicts the crucial influence of high temperature on accelerating the TPC content even if a larger mean sample particle size (i.e., 900 µm) or smaller mean sample particle size (i.e., 500 µm) is employed.

The interaction effect of changes in the time and mean sample particle size on TPC content (Figure 2d) shows that by increasing the time (from 30 min to 90 min) and increasing the mean sample particle size (from 500 µm to 750 µm), maximum TPC may be obtained. However, a longer extraction time (>90 min) with increasing the mean sample particle size (>750 µm) could lead to the reduction of TPC content, highlighting the negative combined influence of longer extraction time and larger mean sample particle size on TPC content.

The interaction effect of changes in time and the solid-to-solvent ratio on TPC content is presented in Figure 2e. By reducing the solid-to-solvent ratio (from 10 g/100 mL to 1 g/100 mL) and increasing the time (from 30 to 120 min), the optimum TPC content (>35 mg GAE/g db) may be achieved, indicating that the positive influence of the solid-to-solvent ratio reduction outweighs the negative effect of long SCW time on TPC.

The interaction effect of changes in the solid-to-solvent ratio and mean sample particle size on TPC content is presented in Figure 2f. Decreasing the solid-to-solvent ratio (from 10 g/100 mL to 1 g/100 mL) with a decrease in mean sample particle size (from 1000 µm to 500 µm) could result in enhanced TPC content. The optimum TPC content (>35 mg GAE/g db) could be attained at a solid-to-solvent ratio of 1 g/100 mL and a mean sample particle size of 500–700 µm.

### 2.3. Antioxidant Activity of the Polyphenolic Extract

**Figure 3:**
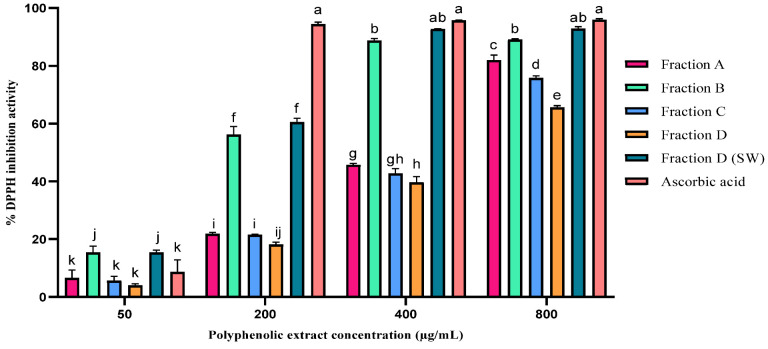
% DPPH radical inhibition activity (% *D_IA_*) of ethanolic extracts (Fractions A, B, C, D) and subcritical water extract (Fraction D (SW)) of apple pomace compared to ascorbic acid. Fraction A (pulp, peel, seed, core, and stem), fraction B (peel), fraction C (seed and core), and fraction D (pulp and peel), data are mean values ± SD from three replicates (*n* = 3); mean values with different letters show significant difference at *p* < 0.05.

The antioxidant activity (% *D_IA_*) of the polyphenolic extracts of different AP fractions obtained using ethanol extraction and SCW was determined at different concentrations and compared with ascorbic acid as the reference compound (Figure 3). The results demonstrate that % *D_IA_* varied among fractions of AP and increased with increasing the extract concentration. The highest % *D_IA_* among the fractions extracted with ethanol was achieved in the polyphenolic extract of fraction B (89.03 ± 0.93%). The data indicate that the peel polyphenolic extract possessed the highest % *D_IA_* in all studied concentrations compared to other fractions (*p* < 0.05). Moreover, the % *D_IA_* of the peel extract can be comparable to the % *D_IA_* of the ascorbic acid at concentrations of 400 and 800 µg/mL. Similarly, Leontowicz et al. showed a % *D_IA_* difference when using different parts of an apple [46]. Notably, the highest % *D_IA_* was achieved at a concentration of 800 µg/mL (*p* < 0.05); however, fraction B extract did not show a significant difference in % *D_IA_* at concentrations of 400 and 800 µg/mL.

Moreover, the results reveal that the % *D_IA_* of the SW extract at a concentration of 400 µg/mL reached 92.69 ± 0.14%, which could be comparable to the % *D_IA_* of the ascorbic acid (94.41 ± 0.74%) obtained at a concentration of 200 µg/mL. The Maillard reaction, which has been reported to happen at high temperatures (i.e., 200 °C) and resulted in the formation of neo-antioxidant compounds [47], is hypothesized to be the one reason for the high % *D_IA_* of the SW extract. Another reason can be due to the high concentration of extracted TPC under SCW conditions.

The half-maximal effective concentration (EC_50_) of the ethanolic extract of AP fractions was obtained at 200.09, 471.65, 508.88, and 584.34 µg/mL for fractions B, A, C, and D, respectively. The lowest EC_50_ value (200.09 µg/mL) was attributed to the extract of peel, which indicated that the peel extract showed the strongest % *D_IA_*, and 50% of % *D_IA_* could be obtained at a lower extract concentration when peel was applied compared to other fractions. Following our study, Giomaro et al. found a lower EC_50_ value of the red apple peel ethanolic extract (0.1 ± 0.02 mg/mL) than that of pulp (0.13 ± 0.01 mg/mL) [27]. A study by Mihailović et al. [48] showed that higher concentrations of polyphenols with potent antioxidant capacities including hyperoside, chlorogenic acid, epicatechin, quercitrin, and catechin can be the reason for higher % *D_IA_* of the Malus sylvestris peel extract compared to the pulp with EC_50_ values of 240 ± 6 µg/mL and 286 ± 7 µg/mL, respectively.

The differences in % *D_IA_* observed among ethanolic extract of AP fractions in this study can be associated with different distributions of polyphenolic compounds with different concentrations, their chemical composition, and the degree of possible oxidation [32]. The higher % *D_IA_* of the peel extract (fraction B) compared to other fractions could be due to the existence of a higher concentration of polyphenolic compounds including quercetin and quercetin glycosides, which are more potent in radical scavenging activity than some other polyphenols such as chlorogenic acid and phloridzin, which were detected in high concentration in seed and core extracts (fraction C) [49,50,51].

In accordance with our study, quercetin glycosides (Quercetin-3-glucoside and Quercetin-3-arabinoside) were shown to be more potent DPPH radical scavengers (EC_50_ = 0.1–0.11 mg/mL) than chlorogenic acid (EC_50_ = 0.24 mg/mL) and phloridzin (EC_50_ = 0.6 mg/mL) in extracts from Gala AP [50]. Chinnici et al. revealed that polyphenolic compounds qualitatively and quantitatively affected the % *D_IA_* of the peel and pulp extracts from Golden Delicious apples. Higher DPPH radical inhibition activity (14.96 ± 2.36 mM Trolox equivalents kg^−1^ fw), more than two-fold, was found in the extract of organic peel compared to the pulp extract (6.28 ± 2.72 mM Trolox equivalents kg^−1^ fw), which was attributed to the presence of a higher concentration of polyphenols such as flavanols (catechin and epicatechin), quercetin glycosides, and procyanidins with higher capacities compared to hydroxycinnamates (chlorogenic acid) and dihydrochalcones (phloridzin) with lower potential in DPPH radical inhibition activity [49]. Nile et al. found that quercetin-3-glucoside extracted from AP displayed the highest % *D_IA_* (90.75%), compared to other polyphenols such as chlorogenic acid, coumaric acid, epicatechin, and phloridzin [51].

### 2.4. HPLC analysis of Polyphenolic Compounds

**Figure 4:**
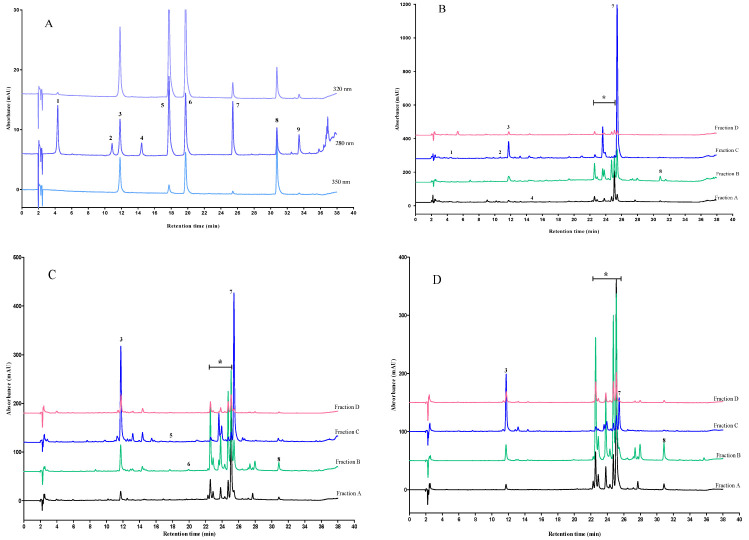
Chromatograms from HPLC of polyphenol standards (**A**) and ethanolic extracts of apple pomace fractions (fractions A, B, C, and D) recorded at (**B**) 𝜆 = 280 nm; (**C**) 𝜆 = 320 nm; (**D**) 𝜆 = 350 nm. Numbers identify the polyphenol peaks: 1: gallic acid; 2: (+)-catechin hydrate; 3: chlorogenic acid hydrate; 4: (−)-epicatechin; 5: p-coumaric acid 6: Ferulic acid; 7: phloridzin hydrate; 8: quercetin; 9: phloretin; (*): unknown quercetin glycosides. The concentration of all the standards was 3.125 µg/mL. Fraction A (pulp, peel, seed, core, and stem), fraction B (peel), fraction C (seed and core), and fraction D (pulp and peel).

HPLC chromatograms and the results of the identified polyphenols are depicted in Figure 4 and Table 4. The concentration of polyphenolic compounds varied among AP fractions and phloridzin, chlorogenic acid, and quercetin were major identified polyphenols. Phloridzin was the main polyphenolic compound with a high concentration in all fractions. Fraction C containing seed and core contained the highest concentration of phloridzin compared to peel, a mixture of peel and pulp, and whole parts of AP. Consistent with the results presented in this study, several other studies reported phloridzin as the most abundant polyphenol in AP from different varieties including Idared and Šampion [37,52,53].

The second most abundant polyphenol identified in all fractions was chlorogenic acid, and the highest concentration was obtained in the extract of fraction C. Chlorogenic acid constituted one of the main polyphenols of the pulp of seven varieties of Crab AP as reported by Górnaś et al. [54]. Quercetin was also found in all the fractions with the highest concentration in fraction B. Kalinowska et al. also detected quercetin in the peel of two apple varieties with different concentrations, which is dependent on the variety and the method of polyphenol extraction. Papierowka and Gold Milenium peels contained quercetin with a concentration of 87.185 ± 6.09 µg/g db extract and 4.76 ± 0.3 µg/g db extract with ultrasound-assisted extraction and 142.40 ± 18.67 µg/g db extract and 14.89 ± 0.83 µg/g db extract with 80% methanol, respectively. However, quercetin glycosides constituted a much higher concentration than quercetin [30].

Another polyphenol that was found in three fractions of A, C, and D was gallic acid. A previous study showed the presence of gallic acid in the peel of two apple varieties of Papierowka and Gold Milenium, but in our study, it was not identified in the peel. Ferulic acid, p-coumaric acid, (+)-catechin, and (−)-epicatechin were also found in AP fractions (Table 4). A previous study found (+)-catechin and (−)-epicatechin in the peel and pulp of 15 varieties of apples; however, these polyphenols were not detected in the peel and pulp in our study, which may be due to their enzymatic oxidation when they were exposed to different processes such as crushing [55].

Moreover, the chromatograms of the fractions demonstrated several major unidentified peaks (specified by * symbol, Figure 4) with retention times of 22.57, 23.80, 24.72, and 25.08 min, which showed maximum UV absorption at 350 nm (Figure 4D). It is hypothesized that these peaks are indicative of the presence of flavonol glycosides such as quercetin glycosides in AP fractions since several studies found quercetin glycosides to be one of the main polyphenols of apples, which were detected mainly in peel [30,53,55,56] and retained in pomace during juice [57] cider [58] and puree production [59]. Moreover, considering the HPLC chromatograms of several other studies, it can be found that quercetin glycosides, due to their sugar moieties, are more hydrophilic (polar) than aglycone (quercetin) and also phloridzin (phloretin glycoside), thus they were eluted earlier than phloridzin and quercetin, and their peaks were close to the phloridzin peak as the order of unidentified polyphenol, phloridzin, and quercetin elution of the present study exhibited the same trend (Figure 4 B–D) [48,56,60]. Therefore, considering previous studies and our observation, it was suggested that four major unknown peaks of the present study could be identified as a different class of quercetin glycosides.

Considering our results, it can be found that the composition and content of polyphenols are different among various studied AP samples. These changes can be attributed to the variation in apple variety, manufacturing procedures, a fraction (part) of AP used as well as different extraction techniques for polyphenol extraction [51,61]. Fraction C contained the highest total concentration of polyphenols compared to other fractions in terms of selected polyphenols for detection; however, as the HPLC chromatograms exhibited, other different polyphenolic compounds were present in the polyphenolic extract of the fractions, particularly in fraction B, which were not among the main polyphenols quantified in this research.

Comparing the results of the determination of polyphenolic compounds with the Folin–Ciocalteu (F-C) assay (Section 2.2) with those obtained from HPLC, differences were observed between the two assays. Based on the F-C result, fraction D contained the highest TPC, while the lowest polyphenol concentration was attained with the HPLC assay in this fraction. It has been reported that non-polyphenolic compounds such as reducing sugars, some vitamins, some amino acids, proteins, and simple inorganic ions could react with F-C reagent and interfere with the result of this assay, and due to the fact that polyphenolics are the most abundant compounds in most plants, the F-C assay has been considered as an acceptable method for polyphenolic content determination [30,62,63]. Therefore, the observed difference in this study between the F-C assay and the HPLC result could be due to the presence of non-polyphenols in AP extracts, which could react with the F-C assay and overestimate the polyphenolic compounds.

### 2.5. Cell Viability of the Polyphenolic Extract

**Figure 5:**
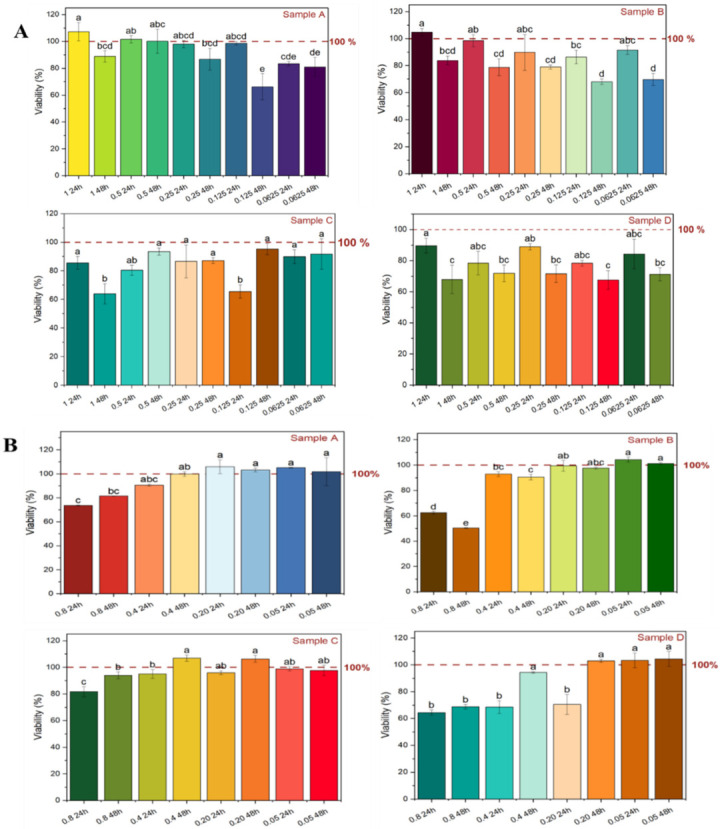
Cell viability of (**A**) fibroblasts 3T3-L1 and (**B**) NS-SV-AC cell lines exposed under ethanolic extract of apple pomace fractions at different concentrations during 24 h and 48 h; sample A (pulp, peel, seed, core, and stem), sample B (peel), sample C (seed and core), and sample D (pulp and peel); control was considered 100%; mean values with different letters show significant difference at *p* < 0.05.

The viability of fibroblast (3T3-L1) and NS-SV-AC cells exposed to different concentrations of ethanolic extract from AP fractions was investigated (Figure 5). The results showed that the polyphenolic extract of fractions did not present a cytotoxic effect on the viability of fibroblast (3T3-L1) cells at the highest employed concentration of 1 mg/mL after 24 h (>80% cell viability) (Figure 5A). Cell viability of extract was shown to be dependent on fraction type, extract concentration, and time. The polyphenolic extract of fractions A and B slightly increased the percentage of viable cells by about 107.27 ± 6.81% and 104.65 ± 2.79%, respectively, at a concentration of 1 mg/mL during 24 h; however, cell viability was decreased in exposure to extracts of fractions C and D, and determined to be 85.57 ± 4.45% and 89.69 ± 4.73%, respectively. The reduction of cell viability was observed after 48 h treatment with the extracts.

The results of the impact of polyphenolic compounds from AP fraction on NS-SV-AC cells showed cytocompatibility of polyphenolic extracts of AP fractions at a concentration of 0.05 mg/mL after 24 h (Figure 5B) in which cell viability was achieved at 105.04 ± 0.27%, 104.38 ± 2.02%, 98.95 ± 1.61 and 103.23 ± 5.52% for extracts of fractions A, B, C, and D, respectively. Increasing the extract concentration from 0.05 mg/mL to 0.8 mg/mL resulted in the reduction of cell viability of about 20%, 50%, 4%, and 34% by employing the extract of fractions A, B, C, and D, respectively, after 48 h. The present work was the first study that exhibited the cytocompatibility of the polyphenolic extracts of AP fractions, which was influenced by the extract concentration and type of AP fraction used [64,65,66]. 

## 3. Materials and Methods

### 3.1. Materials

The four fractions of AP samples with three varieties of Reinette Hernaut, Cox, and Topaz were kindly provided by Materne-Conflux company, located in Namur, Belgium. Each AP fraction was mixed to assure its homogeneity using a laboratory-scale blender and then oven-dried at 60 °C until achieving a constant weight having a moisture content of less than 5%. The dried samples were ground using a lab grinder to a fine powder and then sieved (VWR Chemicals, Belgium) to achieve a particle size of less than 500 µm. The fractions were then kept in a −18 °C freezer.

Acetic acid, sodium carbonate, 2,2-diphenyl-1picrylhydrazyl radical (DPPH), 3-(4,5-dimetylthiazol-2-yl)-5-(3-carboxylmethoxyphenyl)-2-(4-sulfophenyl)-2H-tetrazolium (MTS) and commercial polyphenolic compounds of p-coumaric acid, ferulic acid, quercetin, and gallic acid were provided by Merck Chemical Co. (Darmstadt, Germany). Acetonitrile and commercial polyphenolic compounds of chlorogenic acid hydrate, (+)-catechin hydrate, (−)-epicatechin, phloretin, and phloridzin hydrate were purchased from Tokyo Chemical Industry Co. (Tokyo, Japan). Ethanol and methanol were provided by VWR Chemicals Co. (Belgium). Folin–Ciocalteu’s phenol reagent was purchased from Chemical Lab (Belgium). Dulbecco’s Modified Eagle Medium (DMEM), F12, penicillin-streptomycin, trypsin/ethylenediaminetetraacetic acid (EDTA), fetal bovine serum (FBS), sodium pyruvate, and L-glutamine were provided by Gibco, Invitrogen Corporation (Paisley, UK).

### 3.2. Physicochemical Characterization

To measure the contents of hydrogen, carbon, nitrogen, and sulfur of different fractions of AP, an elemental analyzer (LECO TruSpec CHN, Saint Joseph, Michigan, USA) was utilized. The oxygen content was obtained by subtraction of the combined contents of carbon, hydrogen, nitrogen, sulfur, and ash from the unity value [67]. Then, proximate analysis was carried out to determine the moisture, lipid, protein, carbohydrate, and ash contents of different fractions of AP. Standard methods of ASTM E1756-08 [68] and ASTM D 2017-98 [69] were utilized to determine moisture and ash contents, respectively. AOCS official method Ba4e-93 (AOAC 1998) was applied to determine protein content. The Soxhlet method [70] and Klason method [71] were utilized to determine the lipid and lignin contents, respectively. To determine the carbohydrate content, the combined contents of fat, ash, protein, and lignin were subtracted from the unity value [72].

### 3.3. Extraction Procedure of Polyphenolic Compounds

#### 3.3.1. Ethanol-Water Extraction of Polyphenolic Compounds

The extraction of polyphenolic compounds from four AP fractions was carried out using solvent extraction with ethanol as described in the literature [10,73]. In brief, one g of AP was added to the 80 mL of ethanol 50% *v/v* solution (solid/solvent ratio = 1:80 g/mL) and mixed well with continuous shaking at 200 rpm at 60 °C for 20 min. Then, the supernatants were carefully separated from the residue using a centrifuge at 6000 rpm for 10 min for TPC determination. The lyophilized polyphenolic compound was achieved by concentrating the collected supernatants using a rotary evaporator, freeze-drying, and then preserving at −18 °C.

#### 3.3.2. Subcritical Water Extraction of Polyphenolic Compounds

**Figure 6:**
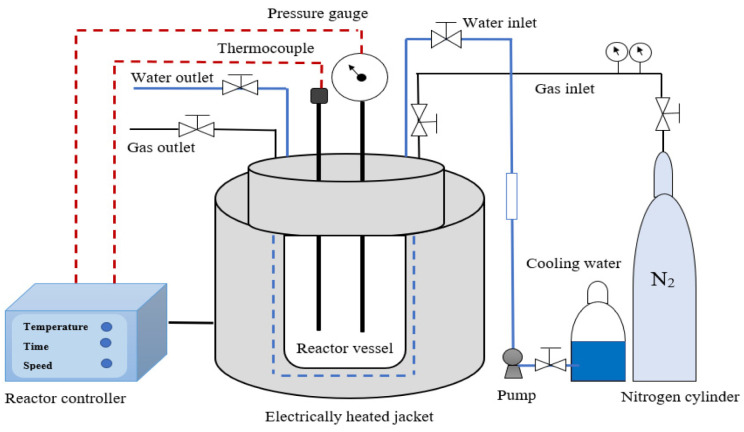
Subcritical water extraction apparatus.

The SCW of polyphenolics from the AP fraction (fraction D) was undertaken using a batch reactor (304 stainless steel autoclave reactor, Henan Lanphan Industry Co., Ltd., Zhengzhou, China) equipped with a PID temperature controller and a heating mantle. Figure 6 presents a schematic of the SCW device used. An optimization study using Box–Behnken design (BBD) was performed to investigate the optimum SCW conditions for enhanced TPC content from AP. For this purpose, the influence of the selected process variables of solid-to-solvent ratio (g/100 mL), mean sample particle size (µm), temperature (°C), and time (min) on TPC yield was investigated. The value ranges of 1–10 g/100 mL, 100–220 °C, 500–1000 µm, and 30–120 min were selected for the solid-to-solvent ratio, temperature, mean sample particle size, and time, respectively. The process parameter ranges were chosen considering the reported values in the literature. The temperature, time, and solid-to-solvent ratio value ranges employed in this research were selected considering the previous studies that examined the SCW of polyphenolic compounds from agro residues [16,43,44,74,75]. The range for mean sample particle size (i.e., 500–1000 µm) was also selected based on a previous study in the literature [39] and was measured using sieves (VWR Chemicals, Belgium). Higher temperatures (i.e., >220 °C) were not considered because of the thermal degradation risk of polyphenolic compounds when such high temperatures are imposed [16,74,75]. The experimental results were statistically analyzed using Minitab^®^ 17.1.0 software.

The conditions of each reaction run were specified in accordance with the experimental design. For each extraction process, the AP sample characterized by specific mass and mean particle size was loaded into the reactor (static mode) and mixed with distilled water to achieve the target solid-to-solvent ratio, following the experimental design. The nitrogen gas was initially injected into the reactor to exclude any trapped air to prevent possible unwanted thermal oxidative reactions, and the SCW extraction was then conducted at a specific temperature for the desired time as specified in the experimental design. The initial pressure was specified as 0.10 MPa (i.e., atmospheric pressure). The pressure for each experimental run changed continuously as the experimental conditions (i.e., temperature, solid loading, etc.) varied in accordance with the experimental (BBD) design. After the extraction was finished, the extract was recovered from the reaction vessel, and filter paper (Whatman paper no.4) was used to separate the solid residue from the extract. Finally, the extract (supernatant) was then analyzed for TPC content via methods discussed in Section 3.4 below. The collected supernatants were then concentrated and then freeze-dried to constant weight and preserved in a freezer at −18 °C.

### 3.4. Measurement of Total Polyphenolic Content (TPC)

The TPC content of the extracts was measured by the Folin–Ciocalteu (F-C) colorimetric method [76]. Briefly, 50 μL from each of the extracts was mixed with 250 μL of 2M F-C reagent and 3 mL of distilled water and vortexed for 10 s. Then, 1 mL of 15% (*w/v*) Na_2_CO_3_ solution was added and the total volume was adjusted to 5 mL with distilled water. The solution was vortexed for 10 s and kept in dark at 25 °C (room temperature). After 1 h incubation, the solution absorbance at the wavelength of 765 nm was measured using a PerkinElmer Lambda 25 UV-vis (Waltham, MA, USA).

Milligrams of gallic acid equivalents (determined using the gallic acid standard curve) per gram of AP (dry weight basis) was defined as TPC content (mg GAE/g db) shown in the following equation (Equation (2)):

(2) $$TPC=\frac{C\times V}{m}$$

where *C*, *V*, and *m* represent the concentration of the sample (mg/mL) achieved from the gallic acid standard curve, volume (mL) of the extraction solvent, and weight (g) of the AP, respectively.

### 3.5. HPLC-DAD/UV Identification of Polyphenolic Compounds

High-performance liquid chromatography equipped with diode-array detection (HPLC-DAD/UV, Agilent, series 1100, Santa Clara, CA, USA) was used to analyze the polyphenolic compounds of AP fractions considering the procedure reported by Zardo et al. [10]. A solution of freeze-dried sample dissolved in dimethyl sulfoxide (DMSO) was prepared at a concentration of 50 mg/mL, filtered through a syringe filter (0.45 μm), and then injected (10 μL) into the HPLC device. The separation was done using a symmetry dC18 column (4.6 × 150 mm, particle size 3 µm, Atlantis ^TM^, Ireland) at a temperature of 20 °C. The binary mobile phase consisting of acetic acid in water 2.5% (*v/v*) (solvent A) and acetonitrile (solvent B) was pumped to the device (flow rate = 1.0 mL/min) considering the gradients of B (3–9%, 0–5 min), (9–16%, 5–15 min), (16–36.4%, 15–33 min), isocratic elution of B (100%, 5 min), and then column reconditioning at 3% of B for 10 min. The detector was set at 280, 320, and 350 nm to simultaneously monitor the different polyphenolic compounds. The retention times and UV spectrum of AP polyphenol extracts were compared with those achieved for commercial polyphenol standards to identify the AP polyphenols. A seven-point calibration curve of external polyphenol standards at a concentration range (1.5625–100 µg/mL) was provided to quantify the polyphenols.

### 3.6. Measurement of the Antioxidant Activity

The antioxidant activity (DPPH radical inhibition activity, % *D_IA_*) of the polyphenolic extract of AP was measured based on a previously reported procedure [77] and compared with ascorbic acid as a reference compound. Different concentrations of freeze-dried extracts were prepared, and their antioxidant activity against DPPH was tested. For this purpose, a solution containing 150 µL of DPPH dissolved in methanol (0.04 mg/mL) and 150 µL of AP extract was prepared and mixed well in a 96-well plate. Then, it was incubated for 30 min in a dark place at room temperature. Finally, the solution absorbance was recorded at 517 nm (Microplate spectrophotometer, Epoch-BioTek, Winooski, VT, USA). The (% *D_IA_*) of the extract was determined using the following equation (Equation (3)):

(3) $$\%D_{IA}=1-\frac{Abs_{s}-Abs_{b}}{Abs_{c}-Abs_{b}}\times 100$$

where *Abs_s_*, *Abs_b,_* and *Abs_c_* represent the sample absorbance (extract + DPPH solution), sample blank absorbance (extract + methanol), and the control absorbance (extraction solvent + DPPH solution), respectively. In addition, to measure the EC_50_ value of the AP extracts, which is defined as the antioxidant concentration needed to quench 50% of the DPPH free radical, interpolation using linear regression analysis was undertaken.

### 3.7. In Vitro Cell Viability

Fibroblast (3T3-L1) cells and human immortalized salivary gland acinar cells (NS-SV-AC) were utilized to investigate the cytocompatibility of polyphenolic extract from AP fractions. For culturing fibroblast (3T3-L1) cells, DMEM supplemented with 10% FCS, 200 U/mL streptomycin, and 200 U/mL penicillin were used. The medium containing DMEM/F12 with 5% FBS and 1% penicillin/streptomycin was employed for culturing NS-SV-AC cells. Cultured cells were incubated at 37 °C in 5% CO_2_. The experiment was performed with cells in passages 7 for fibroblasts (3T3-L1), and between 30 and 38 for NS-SV-AC cells. The cytotoxicity assay was performed by MTS assay as previously reported [78].

Briefly, different concentrations of the sterile AP extracts dissolved in culture medium were exposed to the seeded cells at 5 × 10^3^ cells/well density in 96-well plates, and incubated at 37 °C in 5% CO_2_ for 24 h and 48 h. Then, cells were washed twice with PBS after removing the medium. The MTS solution (100 µL) in culture medium (MTS: culture medium, 1:10 *v/v*) was then added to the well and the plate was incubated for 3 h. Finally, the content of produced formazan was determined spectrophotometrically at 492 nm using a microplate reader (ThermoLab systems, iEMS Reader). The percentage of cell viability of the extract relative to control (*CV%*) was determined as presented below (Equation (4)):

(4) $$CV\%=\frac{Abs_{t}}{Abs_{c}}\times 100$$

where *Abs_t_* represents the absorbance of the treated cells with polyphenolic extracts and *Abs_c_* denotes the absorbance of the control.

### 3.8. Statistical Analysis

The statistical analysis of the data was carried out by GraphPad Prism 9.0. Two-way analysis of variance (ANOVA) using Tukey’s comparison test was employed to assess the difference between the groups. The statistical significance of the data was determined by considering the *p*-value < 0.05.

## 4. Conclusions

This study highlights the utilization of different fractions of industry-sourced apple waste for the recovery of bioactive polyphenolic enriched extracts. The major polyphenols, i.e., phloridzin, chlorogenic acid, and quercetin were identified in different AP fractions. The antioxidant activity (% *D_IA_)* exhibited values comparable to ascorbic acid. Moreover, extracts of AP fractions displayed a different degree of cytocompatibility in fibroblast (3T3-L1) and (NS-SV-AC) cells related to their concentration and the AP fractions employed. The antioxidative property and cytocompatibility of AP extracts as well as enhanced recovery of polyphenolic compounds characterized by a high potential of antioxidant activity achieved using SCW provide important implications for the biological effects of recovered polyphenolic compounds. Understanding this may provide a useful tool for future work in vivo and the possible application of AP polyphenols in biomedical applications such as tissue regeneration and medicinal applications.

## Figures and Tables

**Table 1:** Physicochemical characterization of the different tested fractions of waste apple pomace used in this study.

AP Characterization	Fraction A	Fraction B	Fraction C	Fraction D
Moisture (% *w/w*, wet AP basis)	67.31 ± 1.06	82.77 ± 1.03	62.00 ± 0.35	85.57 ± 1.37
Lipid (% *w/w*, dry AP basis)	1.29 ± 0.52	14.80 ± 0.41	6.77 ± 0.34	5.44 ± 0.04
Carbohydrate (% *w/w*, dry AP basis)	71.94 ± 1.30	65.9 ± 4.54	57.54 ± 5.15	71.77 ± 1.12
Protein (% *w/w*, dry AP basis)	5.94 ± 0.20	4.38 ± 0.00	11.88 ± 0.88	2.50 ± 0.88
Lignin (% *w/w*, dry AP basis)	19.53 ± 1.18	14.48 ± 4.54	23.82 ± 3.92	20.29 ± 1.97
Ash (% *w/w*, dry AP basis)	1.30 ± 0.00	0.44 ± 0.01	1.33 ± 0.05	1.08 ± 0.04
Carbon (% *w/w*, dry AP basis)	46.15 ± 0.64	63.33 ± 0.54	44.42 ± 0.44	43.90 ± 2.83
Hydrogen (% *w/w*, dry AP basis)	6.87 ± 0.11	5.27 ± 0.23	6.64 ± 0.06	6.31 ± 0.11
Nitrogen (% *w/w*, dry AP basis)	0.95 ± 0.03	0.70 ± 0.00	1.9 ± 0.14	0.4 ± 0.14
Oxygen (% *w/w*, dry AP basis)	44.67 ± 0.65	30.18 ± 0.31	45.70 ± 0.46	48.29 ± 2.88
Sulfur (% *w/w*, dry AP basis)	0.07 ± 0.01	0.09 ± 0.01	0.03 ± 0.01	0.03 ± 0.01

Fraction A (pulp, peel, seed, core, and stem), fraction B (peel), fraction C (seed and core), and fraction D (pulp and peel), Data are mean values ± SD from three replicates (*n* = 3).

**Table 3:** The Box–Behnken design (BBD) matrix with experimental response data of TPC content at different process variable conditions was obtained through the SCW of apple pomace (fraction D).

Run	*T*: Temperature (◦C)	*t*: Time (min)	*S*: Mean Sample Particle Size (µm)	*W*: Solid-to-Solvent Ratio (g/100 mL)	*Y*: TPC (mg GAE/g db)
1	100	75	750	10	5.30
2	220	75	500	5.5	18.90
3	100	120	750	5.5	7.19
4	100	75	1000	5.5	6.36
5	160	120	1000	5.5	18.33
6	160	120	750	1	36.26
7	160	75	750	5.5	24.13
8	100	30	750	5.5	6.38
9	220	120	750	5.5	14.02
10	160	75	500	1	36.45
11	160	120	750	10	15.04
12	160	75	1000	1	27.97
13	160	30	750	10	12.19
14	100	75	750	1	15.58
15	220	75	750	10	13.54
16	160	75	1000	10	17.98
17	160	75	750	5.5	23.33
18	160	75	750	5.5	20.10
19	160	30	1000	5.5	18.01
20	220	75	750	1	39.01
21	160	30	500	5.5	18.45
22	160	75	500	10	16.44
23	100	75	500	5.5	6.63
24	160	30	750	1	27.77
25	220	30	750	5.5	20.42
26	220	75	1000	5.5	15.62
27	160	120	500	5.5	16.56

**Table 4:** Quantification of individual polyphenols of ethanolic extract from apple pomace fractions.

Polyphenolic Compounds	Concentration of Polyphenol in Apple Pomace Fractions (mg/100 g db Extract)			
	Fraction A	Fraction B	Fraction C	Fraction D
Phloridzin *	33.02 ± 1.04	167.48 ± 12.93	856.87 ± 17.94	18.17 ± 0.46
Chlorogenic acid *	6.89 ± 0.65	26.37 ± 1.13	97.05 ± 2.45	17.26 ± 0.66
Quercetin	6.42 ± 0.44	22.10 ± 1.88	0.81 ± 0.36	1.84 ± 0.45
Gallic acid	0.91 ± 0.39	nd	1.36 ± 0.37	0.84 ± 0.20
p-coumaric acid	0.22 ± 0.02	nd	0.22 ± 0.06	0.23 ± 0.11
Ferulic acid	0.2 ± 0.04	0.61 ± 0.03	nd	nd
(+)-catechin *	nd	nd	6.82 ± 4.17	nd
(−)-epicatechin	2.98 ± 0.67	nd	nd	nd
Phloretin	nd	nd	nd	nd

All values are expressed as the mean ± standard deviation (n = 4). nd = not detected. * Phloridzin as phloridzin hydrate; chlorogenic acid as chlorogenic acid hydrate; (+)-catechin as (+)-catechin hydrate. Fraction A (pulp, peel, seed, core, and stem), fraction B (peel), fraction C (seed and core), and fraction D (pulp and peel).

## Data Availability

All data generated or analyzed during this study are included in this published article.
